# The Potential Protective Effects of Intermittent Fasting Against Radiation-Induced Brain Damage in a Rat Model: Suggested Involvement of IRS-1/PI3 K/AKT and BDNF/TrkB Signaling Pathways

**DOI:** 10.1007/s12035-025-05059-1

**Published:** 2025-07-01

**Authors:** Eman F. S. Taha, Hebatallah E. Mohamed, Lobna M. Anees, Hayam Mostafa, Eman S. Eldin

**Affiliations:** 1 Health Radiation Research Department, National Center for Radiation Research and Technology, (NCRRT), Egyptian Atomic Energy Authority (EAEA), Cairo, Egypt; 2 Radiation Biology Department, National Center for Radiation Research and Technology, (NCRRT), Egyptian Atomic Energy Authority (EAEA), Cairo, Egypt

**Keywords:** Ionizing radiation, Intermittent fasting, BDNF/TrkB, IRS-1/PI3 K/AKT, GFAP

## Abstract

Fasting has emerged as a promising therapeutic strategy for neurological disorders, offering protection against insults such as ionizing radiation (IR), which can cause irreversible brain damage. Intermittent fasting (IF), including alternate-day fasting (ADF) and time-restricted feeding (TRF), is being explored for its neuroprotective effects with potential involvement of key signaling pathways such as IRS-1/PI3K/AKT and BDNF/TrkB. Thirty-six male Wistar albino rats were randomly divided into six groups: normal feeding (NF, ad libitum feeding), ADF, TRF (6-h feeding window), NF plus radiation (NF-irradiated, 20-Gy cranial exposure), ADF plus radiation (ADF-irradiated), and TRF plus radiation (TRF-irradiated). Oxidative stress markers, antioxidant enzymes, liver and kidney function parameters, and gene/protein expression levels (IRS1, AKT1, PI3K, GFAP, 8-OHdG, BDNF, TrkB) were evaluated using enzyme-linked immunosorbent assay (ELISA) and RT-PCR, complemented by histopathological analysis. IR significantly impaired antioxidant defenses (GSH, GST, CAT), suppressed IRS-1/PI3K/AKT and BDNF/TrkB signaling, and elevated oxidative damage markers (MDA, ROS, 8-OHdG), inflammation (GFAP), and markers of organ dysfunction (ALT, AST, GGT, urea, creatinine). Both IF regimens mitigated these effects; however, TRF demonstrated greater efficacy than ADF. TRF more effectively reduced oxidative stress, improved antioxidant enzyme activity, and more robustly restored metabolic and neurotrophic signaling pathways. Both ADF and TRF provided neuroprotection against radiation-induced brain injury, but TRF exhibited superior outcomes in reducing oxidative stress and preserving neuronal integrity. These findings highlight TRF as a potentially more effective dietary strategy for mitigating radiation-induced neurotoxicity, with possible contributions from the modulation of IRS-1/PI3K/AKT and BDNF/TrkB pathways.

## Introduction

Radiation affects the human body indirectly and directly. Indirectly it may create ions and free radicals that interact with cellular components, directly it may also damage important biological molecules [1]. The brain is susceptible to severe oxidative assaults owing to its rich content of lipids, great energy requirements, and low antioxidant aptitude. Through oxidative alterations in the brain, reactive oxygen species (ROS) raise vulnerability to neuronal damage and functional deficits in neurodegenerative diseases [2]. Furthermore, the brain is an appropriate substrate for peroxidation reactions due to its high concentration of unsaturated fatty acids [3]. Radiation-induced brain damage is caused by a variety of intricate processes, the most prominent of which are oxidative stress, inflammation, and DNA damage [4].

Oxidative stress in the brain is a result of an overabundance of free radicals and ROS, which during normal physiological conditions are efficiently countered by antioxidant mechanisms. Oxidative stress occurs in the brain once this equilibrium is disturbed. Major contributors to antioxidant mechanisms include enzymes such as glutathione peroxidase (GSH-Px), superoxide dismutase (SOD), and catalase (CAT) [5]. In brain cells, the cellular detoxification of ROS depends on GSH system. In neurological illnesses, oxidative stress has been linked to a compromised GSH system in the brain [6]. In many tissues and organs, oxidative stress can permanently damage cellular protein structures, membrane lipids, and DNA. The most prevalent free radical–derived form found in nuclear and mitochondrial DNA is 8-hydroxy2′-deoxyguanosine (8-OHdG), which indicates the extent of oxidative stress–induced damage to cellular DNA [7]. Several processes are involved in oxidative stress in the brain, including redox signaling, neurotransmitter auto-oxidation, and lipid peroxidation. Lipid peroxidation is a multi-step process that can cause brain cell damage because it involves the production of free radicals, oxidation, and chain reaction propagation [8]. Moreover, pathological changes, neuro-function, and aging are all strongly correlated with oxidative stress in the brain. Research has indicated that redox signaling is crucial for the release of neurotransmitters, aging processes, and cognitive function [9].

It has been demonstrated that IF can improve health status and prolong lifespan in mammals [10]. Fasting induces a modified metabolic state that improves the neuron’s bioenergetics, plasticity, and resilience in a way that may alleviate a variety of neurological disorders. Metabolic syndrome, a main risk factor for a diversity of neurological diseases in both humans and animals, is treated and prevented with fasting [11]. Fasting most likely inhibits the development of tumors, may even cure existing tumors, and enhances the way chemotherapy works on malignancies in animals. Fasting reduces the side effects of chemotherapy in human tumors, particularly brain cancers, and may protect healthy cells from the harmful effects of chemotherapy. In animal models, fasting diminishes brain damage, improves cognition, delays age-related cognitive decline, typically slows neurodegeneration, improves functional recovery following a stroke, and alleviates the pathological and clinical characteristics of multiple sclerosis and epilepsy [12]. In IF, there are primarily two types of dietary interventions: time-restricted feeding (TRF), which is more common, and alternate-day fasting (ADF). Alternate day fasting, which can be done multiple times a week, is a 24-h fast followed by a 24-h feeding phase. Time-restricted or early TRF regimens utilize different fasting periods that might last 16, 18, or more hours [13]. Additionally, IF has been shown in recent research to have positive effects on aging brains or various models of disorders affecting the central nervous system (CNS) [14].

Mechanistically, IF augments neuronal resistance to various types of injuries by eliciting a mild adaptive stress response, therefore improving learning, memory, and brain plasticity, while decreasing inflammation and neurodegeneration [15]. IF triggers antistress reactions thus offering protection against oxidative stress and inflammation [16]. Neurotrophins, such as brain-derived neurotrophic factor (BDNF), play vital roles in neuronal differentiation, cell survival, and synaptic function in the central and peripheral nervous systems. Tropomyosin receptor kinase B TrkB, a high-affinity receptor for BDNF, is known to stimulate a variety of intracellular signaling which affects neurogenesis, cell viability, synaptic function, and cognitive function, all of which are involved in physiological and pathological neuronal aspects [17].

Numerous investigations have indicated that the pathophysiology of neurodegenerative diseases may involve modified BDNF/TrkB signaling expression and activity [18]. The BDNF/TrkB pathway activates multiple intracellular signaling cascades such as ERK and PI3 K/Akt [17]. Insulin and insulin receptors are crucial in controlling brain functions, in addition to being involved in synaptic plasticity, learning, and cognitive functions [19]. Insulin receptors are abundantly expressed in neurons, mainly in the hippocampus [20]. The PI3 K/Akt signaling pathway is triggered by insulin’s binding to insulin receptors [21].

Numerous receptors and intracellular signaling molecules trigger PI3 K, which then promote the phosphorylation of serine/threonine kinase (AKT) and induce metabolism, growth, proliferation, and other cellular functions [22]. It has been established that the PI3 K/Akt signal pathway is crucial in the etiology of neurological disorders [23]. Numerous investigations have demonstrated that the PI3 K/Akt pathway may impact brain growth and control several neurological diseases through controlling autophagy and apoptosis, differentiation and proliferation of neuronal cells, circuits, and synaptic plasticity [24]**.**

Therefore, this study is designed to evaluate the effectiveness of intermittent fasting against radiation-induced brain damage in a rat model, with a focus on the potential involvement of the IRS-1/PI3 K/AKT and BDNF/TrkB signaling pathways.

## Materials and Methods

### Animal Collection

Thirty-six male Wistar albino rats at 6–12 weeks of age with a mean weight of 150–200 g were obtained from a breeding unit maintained in the animal house of the National Center for Radiation Research and Technology (NCRRT), Cairo, Egypt. Rats were randomly assigned into six experimental groups, six rats each. The animals collected were housed in well-ventilated wired plastic metabolic cages in the animal facility of NCRRT. The rats were maintained under a standard room temperature (25 ± 2 °C), humidity (60 ± 10%), and a 12/12-h light/dark cycle with free access to a standard chow diet (23% protein) and water [25]. They were allowed to acclimatize for 1 week before the commencement of the experiments; the weight of the animals was estimated at procurement, during acclimatization, at the commencement of the experiment, and every week throughout the experiments using an electronic analytical precision balance.

### Ionizing Gamma (ℽ) Radiation

A radiation resource for ^60^CO (Atomic Energy of Canada Limited; Sheridan Science and Technology Park, Mississauga, Ontario, Canada) supported a cell-40 irradiation unit at the NCRRT, Cairo, Egypt. Rats were whole-brain irradiated with a single fraction of 20 Gy (50% isodose) as mentioned by Shaw, Robbins [26] at a dose rate of 0.701 KGy/h, while anesthetized using ketamine (90 mg/kg ip, Ketalar, Pfizer) and xylazine (10 mg/kg ip, Alfazyne) and placed in a plastic sample dish [27]**.**

### Experimental Groups

**Fig. 1:**
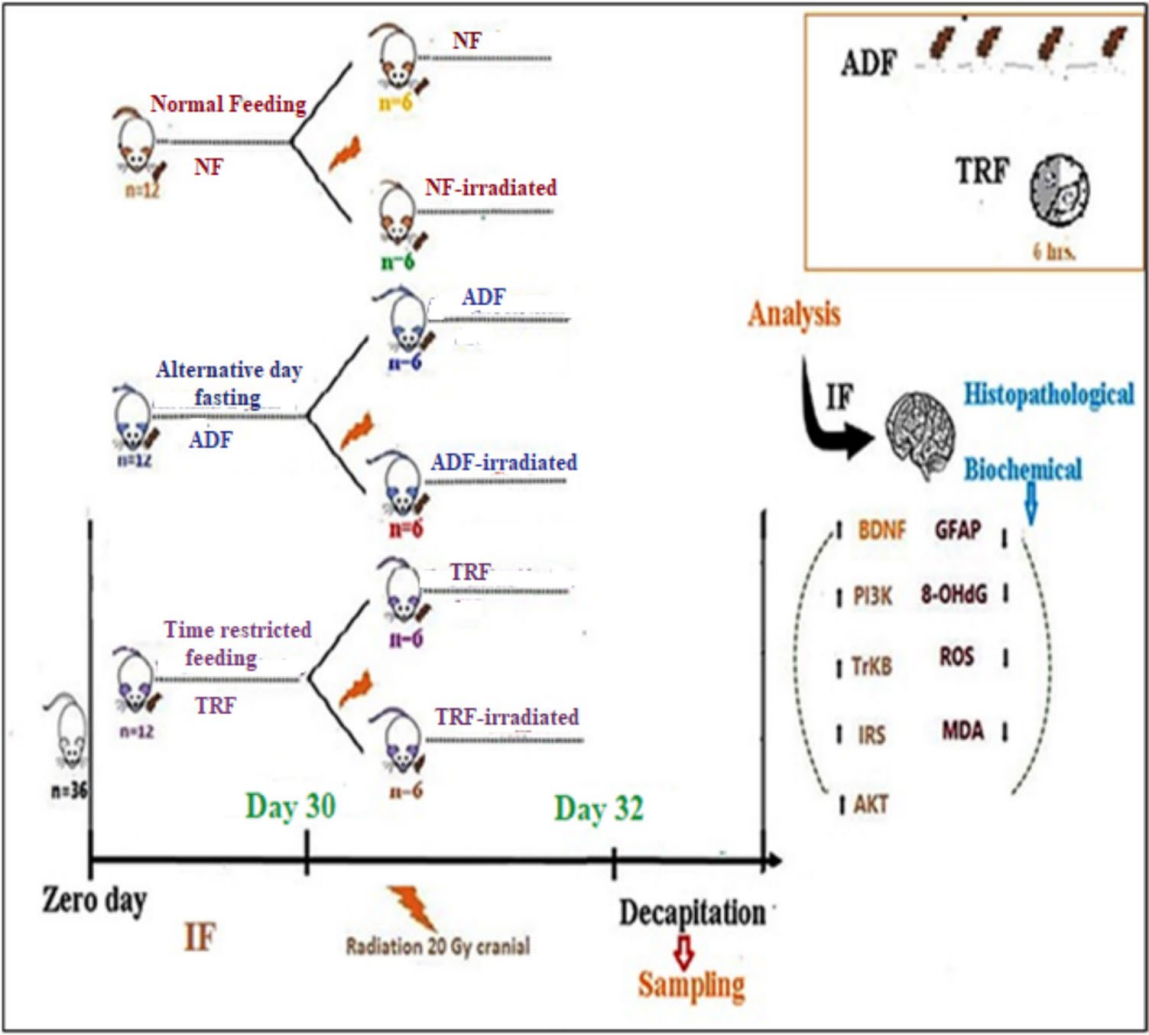
Experimental group design for intermittent fasting and normal feeding regimen

Thirty-six male Wistar albino rats were randomly divided into six experimental groups (*n* = 6 per group). *The Normal Feeding* (*NF*) *group* was maintained on ad libitum feeding for 1 month. *The ADF group* underwent an alternate-day fasting regimen, while *the TRF group* followed a time-restricted feeding schedule for the same period [28]. Three additional groups were subjected to cranial irradiation (20 Gy) following the same one-month feeding protocols: *NF-irradiated*, *ADF-irradiated*, and* TRF-irradiated*. Details of the specific feeding regimens are provided in the following section and illustrated in Fig. 1.

### Feeding Protocols

The feeding regimens were designed to reflect established intermittent fasting patterns. In the ADF groups, rats were given access to food from 9:00 AM to 9:00 PM on feeding days, followed by a complete 24-h fasting period the next day. For the TRF groups, animals received food daily within a restricted 6-h window, from 9:00 AM to 3:00 PM, followed by an 18-h fasting period [28–30]. Throughout the study, all animals had ad libitum access to water.

### Animal Dissection and Sample Collection and Preparation

At the end of the experimental period, rats were deeply anesthetized with an intraperitoneal injection of ketamine (90 mg/kg). While under deep anesthesia, blood samples were collected via cardiac puncture into plain tubes. The collected blood was centrifuged at 3000 × *g* for 5 min, and the resulting serum was stored at − 20 °C for subsequent biochemical analysis [31]. Immediately after blood collection, animals were euthanized by decapitation. Two days after radiation exposure, brains were carefully extracted by craniotomy. Each brain was weighed and then bisected into two hemispheres. One hemisphere was fixed in 10% formal saline for histopathological analysis, while the other was used for biochemical and gene expression studies. For biochemical analysis, the tissue was homogenized in ice-cold phosphate-buffered saline (10 mmol/L, pH 7.4) containing 0.15 M KCl (10% w/v), and the homogenate was centrifuged at 10,000 × *g* for 15 min at 4 °C. The resulting supernatants were stored at − 80 °C until analysis [32]. Additionally, a portion of brain tissue was immediately collected after euthanasia and snap-frozen in liquid nitrogen to preserve RNA integrity. Frozen tissues were pulverized on dry ice using a pre-chilled mortar and pestle, then transferred into tubes containing TRIzol reagent (1 ml per 50–100 mg of tissue) for RNA extraction. Homogenization was performed using a glass-Teflon or Polytron homogenizer at low speed to minimize foaming. The homogenate was then centrifuged at 12,000 × *g* for 10 min at 2–8 °C to remove insoluble debris [33, 34].

### Assessment of Body Weight and Brain Weight Index

The body weight of rats in all groups was recorded on the first day of the experiment (day 0) and subsequently at the end of each week throughout the 4-week experimental period. Body weight changes were monitored weekly to assess the effects of the dietary interventions and radiation exposure over time. The percentage of body weight change (%BWC) was calculated at each time point relative to the previous week’s weight, using the following formula, according to the methodology outlined by Abou Bakr et al. [35]: % BWC = {(Final body weight – Initial body weight)/Initial body weight × 100}, where the initial body weight corresponds to the body weight recorded at the beginning of the study (day 0), and the final weight refers to the body weight at each respective week. This approach allowed for the assessment of weekly changes rather than cumulative change from baseline. For brain weight assessment, the whole brains of experimental rats were surgically removed two days after the final radiation exposure. Brains were rinsed with ice-cold physiological saline, and blotted dry, and their absolute weights were determined following the protocol described by Alimba et al. [36].

### Enzyme-Linked Immunosorbent Assay

ELISA assay kits were used for the determination of GFAP (Cat. No.: MBS2505953, MyBioSource, San Diego, CA, USA), 8-OHdG (Cat. No: K4160-100, BioVision, USA), IRS1 (Cat. No.: MBS029127, MyBioSource, USA), AKT1 (Cat. No.: LS-F49321, LifeSpan BioScience, Inc.), and PI3 K (Cat. No.: MBS260381, MyBioSource, San Diego, CA, USA) levels in the brain tissue homogenates according to the manufacturer’s instructions.

### Quantitative Real-Time PCR of BDNF and TrkB

Reverse transcription-polymerase chain reaction (RT-PCR) was used to determine the gene expression levels of BDNF and TrkB in the brain tissues. RNA was extracted from the tissue samples using a commercially available RNA extraction kit (Invitrogen; Thermo Fisher Scientific, Inc., Waltham, MA, USA), following the manufacturer’s instructions. A spectrophotometer was used to assess the purity and concentration of the RNA. As directed by the supplier, cDNA was produced using the Bio-Rad iScript cDNA Synthesis Kit. Forward and reverse primer sequences used for quantitative RT-PCR, or BDNF (Rat Untagged Clone–RN216152, OriGene Technologies, Inc.), and TrkB (Rat Tagged ORF Clone–RR206400, OriGene Technologies, Inc.) are presented in Table 1. The amplification was performed through two-step cycling (95–60 °C) for 45 cycles in a Light Cycler 480 Instrument RT-PCR Detection System (Roche) following the supplier’s instructions. All samples were assayed in triplicate. The − ΔCt technique was employed to calculate gene expression. To standardize the results, glyceraldehyde-3-phosphate dehydrogenase (GAPDH) was used as a reference gene.

**Table 1:** Forward and reverse primer sequences for primers used for quantitative RT-PCR analysis

Gene	Primer sequence	Accession number
BDNF	F:5′-ATGACCATCCTTTTCCTTAC-3′ R:5′-CTATCTTCCCCTTTTAATGG-3′	NM_001270638
TrkB	F:5′-ATGTCGCCCTGGCCGAGGTG-3′ R:5′-GCCTAGGATG TCCAGGTAGA-3′	NM_012731
GAPDH	F:5′-TGGATTTGGACGCATTGGTC-3′ R:5′-TTTGCACTGG TACGTGTTGAT-3′	XM_032887061

### Estimation of Biochemical Parameters

The serum samples were analyzed to assess various biochemical markers for liver function tests, including serum alanine aminotransferase (ALT) and aspartate aminotransferase (AST), using the method described by Reitman and Frankel [37] with catalog number AT 10 34 (Bio-diagnostic kit, Giza, Egypt). The determination of serum gamma-glutamyl transferase (GGT) levels was carried out using the Szasz method [38] and a kit obtained from the Egyptian Co. for Biotechnology, Spectrum Diagnostics S.A.E. Kidney function was evaluated by measuring creatinine and urea levels. The method outlined by Bartles et al. [39] was employed for creatinine analysis (Cat. No. CR 1251), while the Fawcett and Scott [40] method was utilized for urea analysis (Cat. No. UR 2110). Both measurements were performed using a colorimetric method with the assistance of a commercial kit (Bio-diagnostic kit, Giza, Egypt). The assays were conducted according to the instructions provided in the respective kit manuals, ensuring the generation of accurate and reliable results.

### Assessment of Oxidative Stress Markers

The levels of oxidative stress (OS) in brain homogenates were evaluated using commercial kits from the Bio Diagnostic Company. The assessment involved measuring the levels of malondialdehyde as a lipid peroxidation marker (MDA; MD 2529), as well as the activities of catalase (CA 2517) and glutathione reduced (GSH; GR 2511). The manufacturer’s instructions were followed in conducting these measurements.

### Assessment of GST Activity

The GST activity was assayed spectrophotometrically at 25 °C with reduced glutathione (GSH) and 1-chloro-2, 4-dinitrobenzene (CDNB) as substrates. This was done by watching an increase in absorbance at 340 nm. For each assay, 1 ml of assay cocktail was prepared (980 μl PBS pH 6.5, 10 μl of 100 mM CDNB, and 10 µl of 100 mM GSH), then 100 µl of cocktail was removed and the remaining 900 µl was placed into a 1.5-ml cuvette. To zero the spectrophotometer, 1 ml of distilled water was used, and to the blank cuvette 100 μl PBS was added to 900 μl of cocktail and absorbance was measured at 340 nm, every 1 min, for 3 min. To the test cuvette, 100 μl of sample was added to 900 μl cocktail, mixed, and absorbance was measured at 340 nm as the above [41].

### Quantification of Reactive Oxygen Species

A modified version of a previously described assay for the intracellular conversion of nitro blue tetrazolium (NBT) to formazan by superoxide anion was used to measure the reactive oxygen species (ROS) in the resulting supernatant of brain tissue homogenates from both control and experimental group as described in the studies by Vrablic et al. [42] with slight modification. Briefly, 200 µL NBT (1.00 mg/ml) was added to the homogenates, followed by additional incubation for 1 h at 37 °C. The solutions were then treated with 100 µL KOH (2 M). The absorbance at 570 nm was determined spectrophotometrically and expressed as µmol NBT reduced/g tissue.

### Histopathological Processing

Brain tissue specimens were collected from all animal groups and then fixed in 10% neutral buffered formalin. The fixed samples underwent trimming, washing, and dehydration in increasing alcohol grades, then cleaned in xylene, paraffin-embedded, sectioned at 4–6 μm thickness, and stained with hematoxylin and eosin, according to Bancroft and Gamble [43]. Stained slide sections were then viewed under a light digital microscope (Olympus xc30, Tokyo, Japan). An absolute neuronal cell counts as well as a cell count of all ischemic damaged neurons was done using ImageJ/Fiji v 1.50 (ImageJ Software downloaded at https://imagej.nih.gov/ij). The ratio of damaged neurons to the complete neuronal cell count was graded into five categories (1 = 0–20%, 2 = 20–40%, 3 = 40–60%, 4 = 60–80%, and 5 = 80–100%). For each animal, four hippocampal subregions (CA1, CA3, dentate gyrus, and subiculum) and cerebral cortex were evaluated independently, and their respective scores were summed to yield an overall neuro histopathological severity score [44].

### Statistical Analysis

Statistical analyses were performed using GraphPad Prism 9.5.1 (Graphpad Software Inc., San Diego, CA, USA). Raw data were statistically analyzed for the normal distribution using the Shapiro–Wilk test. The data were expressed as the mean ± SE (*n* = 6). Statistical analysis was performed using two-way analysis of variance (ANOVA) to assess the effects of IF (ADF and TRF) and radiation (NF non-irradiated and NF-irradiated) and their interaction on the measured parameters. Post hoc comparisons were conducted using Tukey’s test. A *p*-value < 0.05 was considered statistically significant. The number of rats used per group was calculated using GPower 3.1.9.4 (Heinrich Heine University Düsseldorf, Dusseldorf, Germany) [45]. Taking into consideration our preliminary results, we have considered an effect size of 1.15. Based on significance level (α) at 0.05 and testing power (1-β) at 0.8 for six groups, a total of 36 adult male Wistar rats was determined (six rats/group).

## Results

### Effects of Alternate-Day Fasting and Time-Restricted Feeding on Rats’ Body Weight Following Radiation Exposure

The results demonstrated the effects of ADF and TRF protocols on body weight (BW) and brain weight in rats exposed to radiation. There were no significant differences in initial body weight (IBW) among the experimental groups (Table 2, *p* > 0.05). However, final body weight (FBW) was significantly reduced in the ADF group compared to both the NF and NF-irradiated groups, while the TRF group did not differ significantly from the NF group. Regarding the percentage of body weight change (%BWC), both ADF and TRF groups exhibited significant reductions compared to NF (*p* < 0.05), suggesting a weight-lowering effect of intermittent fasting.

**Table 2:** Changes in body weight, body weight gain, and absolute brain weight in all experimental rats

	Non-irradiated				Irradiated	
	NF	ADF	TRF	NF	ADF	TRF
IBW (g)	154.4 ± 1.3	151. ± 3.1	150.8 ± 5.4	151.4 ± 2.2	154.4 ± 3.9	150.6 ± 4.1
FBW (g)	225.4 ± 3.6	182.8 ± 7.6^ab^	199.0 ± 9.1^ab^	225.2 ± 4.4	190.2 ± 5.4^ab^	209.8 ± 7.9
% BWC	45.98 ± 1.8	20.52 ± 3.9^ab^	31.81 ± 1.8^ab^	48.8 ± 3.2	23.2 ± 2.8^ab^	39.2 ± 3.2^c^
Brain weight (g)	1.1 ± 0.1	1.2 ± 0.08	1.1 ± 0.03	1.4 ± 0.07	1.1 ± 0.06^a^	1.2 ± 0.07

*BW* body weight, *FBW* final body weight, *IBW* initial body weight; percentage of body weight change (% BWC) = (FBW-IBW)/IBW × 100. Data were presented as mean ± SE (*n* = 6). Data were statistically analyzed using a two-way analysis of variance (ANOVA) followed by a post hoc Tukey test

^a^Significant vs the NF group

^b^Significant vs the NF-irradiated group

^c^Significant between ADF-irradiated and TRF-irradiated groups at *p* < 0.05

A two-way ANOVA was conducted to evaluate the effects of dietary regimen and radiation exposure on %BWC in rats across 4 weeks. The analysis revealed significant main effects of dietary regimen (*F*(3, 48) = 303.7, *p* < 0.0001) and radiation exposure (*F*(5, 48) = 34.6, *p* < 0.0001), as well as a significant interaction between dietary regimen and radiation (*F*(15, 48) = 8.213, *p* < 0.0001), indicating that the impact of dietary interventions on body weight was modulated by radiation status.

**Fig. 2:**
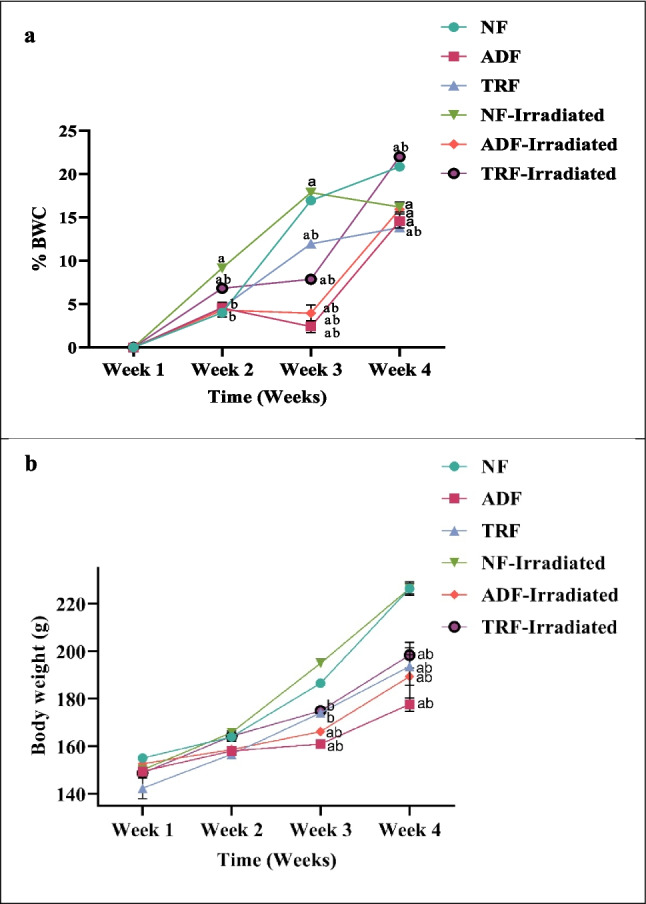
Effect of intermittent fasting (ADF and TRF) on **a** weekly percentage change in body weight (%BWC) and **b** absolute body weight (g) across the 4-week experimental period in all experimental rat groups. Week 1 represents the baseline measurement prior to the initiation of fasting interventions, while weeks 2–4 indicate the changes observed at the end of each fasting week. %BWC was calculated relative to the body weight recorded at the end of the previous week (i.e., not relative to baseline). Data were presented as mean ± SE (*n* = 6). Statistical analysis was performed using repeated measures one-way ANOVA followed by Tukey’s post hoc test.^a^Significant vs. NF group. ^b^Significant vs. NF-irradiated group. ^c^Significant between ADF-irradiated and TRF-irradiated groups (*p* < 0.05)

Post hoc Tukey’s analysis showed that during the first 2 weeks, there were no significant differences in % BWC among the groups, including between ADF and TRF (week 1: mean difference = 7.1, *p* = 0.6135; week 2: mean difference = 1.4, *p* = 0.9995). By week 3, ADF rats exhibited a significantly lower %BWC compared to the NF group (mean difference = 25.5, *p* < 0.0001), while the difference between TRF and NF did not reach statistical significance (mean difference = 12.5, *p* = 0.0770). The comparison between ADF and TRF in week 3 approached significance (mean difference =  − 13.1, *p* = 0.0571), suggesting a trend toward greater weight reduction with ADF (Fig. 2a).

By week 4, both ADF and TRF groups demonstrated significantly lower % BWC compared to NF (ADF: mean difference = 48.6, *p* < 0.0001; TRF: mean difference = 32.6, *p* < 0.0001). Importantly, ADF resulted in a significantly greater reduction in % BWC than TRF at this time point (mean difference =  − 16.0, *p* = 0.0102). These results indicate that while both intermittent fasting regimens were effective in mitigating body weight gain, ADF produced a more pronounced effect than TRF, particularly in the later stages of the study (Fig. 2a).

No significant differences in absolute brain weight were observed among any of the groups (*p* > 0.05), indicating that neither dietary regimen nor radiation exposure affected brain mass.

In summary, these findings demonstrate that both ADF and TRF significantly reduced body weight gain compared to controls, with ADF showing a superior effect by the fourth week. The significant interaction between dietary regimen and radiation exposure further highlights the potential of intermittent fasting, especially ADF, as a protective strategy against radiation-induced metabolic disturbances.

### Effects of Alternate Day Fasting and Time-Restricted Feeding on Oxidative Stress Markers, and Antioxidant Enzymes in Irradiated and Non-irradiated rats

**Fig. 3:**
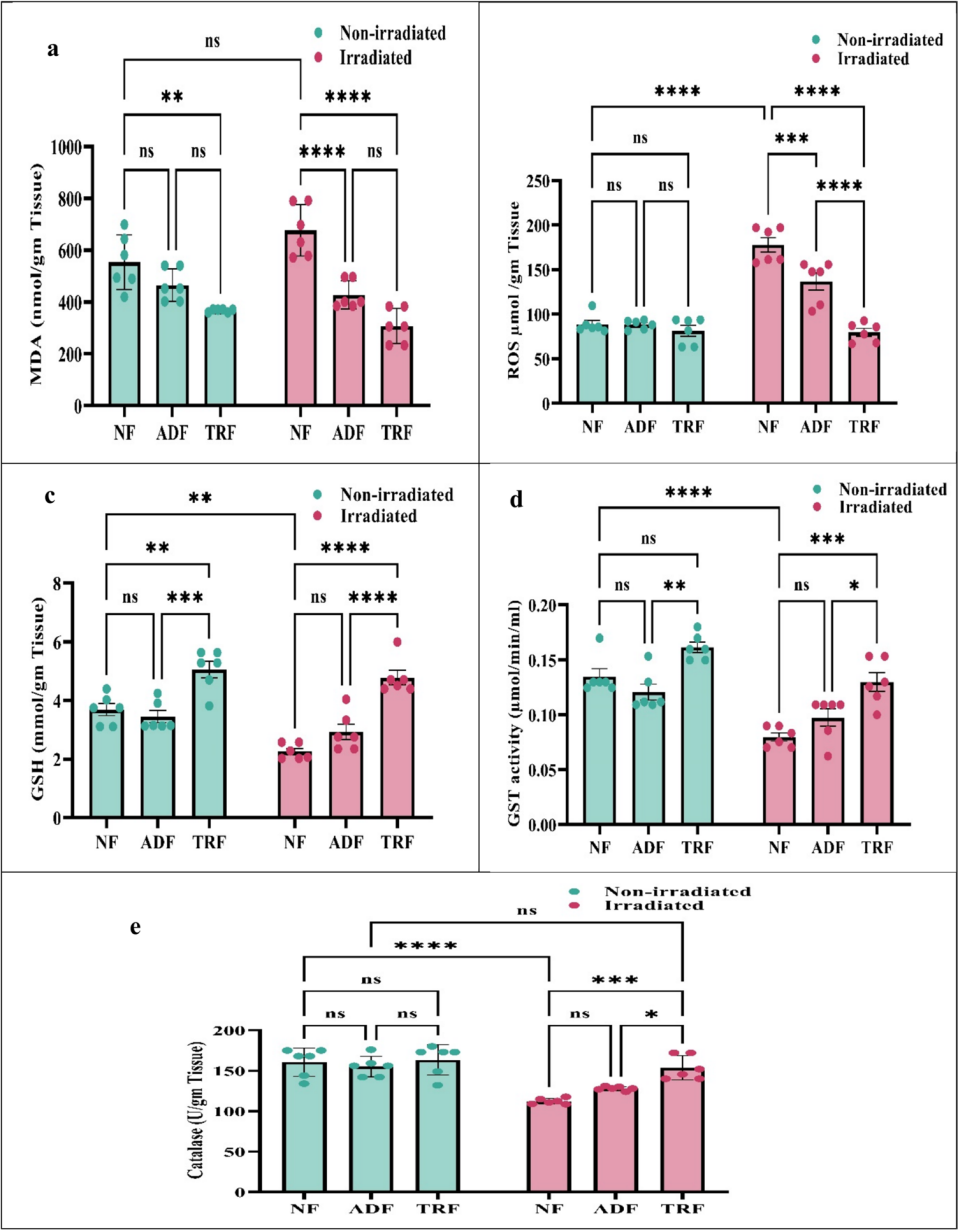
Impact of normal feeding (NF), alternate day fasting (ADF), and time-restricted feeding (TRF) interventions for 4 weeks on oxidant/antioxidant (MDA and ROS/GSH, GST, and catalase) levels in the brain tissue of irradiated and non-irradiated rats. The values were given as the mean ± SE (*n* = 6). Statistical analysis was performed using two-way ANOVA followed by Tukey multiple comparison test at *p* < 0.05. ns: non-significant; *: significant at *p* < 0.05 level; **: significant at *p* < 0.01 level; ***: significant at *p* < 0.001 level; and****: significant at *p* < 0.0001 level

Data represented that radiation exposure led to a pronounced increase in oxidative stress, as evidenced by significant elevations in both malondialdehyde (MDA) and reactive oxygen species (ROS) levels. Two-way ANOVA revealed significant interactions between diet and radiation for both MDA (*F*(2, 30) = 5.4, *p* = 0.0094) and ROS (*F*(2, 30) = 26.7, *p* < 0.0001), indicating that the effect of dietary intervention on these oxidative markers was dependent on irradiation status. In the NF group, irradiation caused a marked increase in MDA and ROS compared to non-irradiated controls. However, both ADF and TRF regimens significantly mitigated these increases, with TRF demonstrating a more substantial reduction in oxidative damage. Post hoc Tukey’s tests showed that NF-irradiated rats had markedly higher levels of both MDA and ROS compared to all other groups (e.g., for MDA: vs. TRF-irradiated, 369.1, *p* < 0.0001; for ROS: vs. TRF-irradiated, 98.1, *p* < 0.0001). Both ADF-irradiated and TRF-irradiated groups exhibited significantly reduced MDA and ROS levels relative to NF-irradiated rats (all *p* < 0.001). Furthermore, TRF-irradiated rats had the lowest levels of both markers, and ROS was significantly lower in TRF-irradiated than in ADF-irradiated rats (57.1, *p* < 0.0001), while MDA did not differ significantly between the two fasting regimens (mean difference = 119.9, *p* = 0.0798) (Fig. 3a, b).

In parallel, the endogenous antioxidant defense system, represented by glutathione (GSH), glutathione S-transferase (GST), and catalase, was significantly compromised following radiation exposure, particularly in the NF group. Two-way ANOVA showed significant interactions for GSH (*F*(2, 30) = 3.6, *p* = 0.0392) and catalase (*F*(2, 30) = 6.4, *p* = 0.0048), and strong main effects of both diet and radiation for all three enzymes.

Post hoc Tukey’s tests demonstrated that radiation significantly reduced catalase activity in NF-irradiated rats compared to NF non-irradiated controls (48.3, *p* < 0.0001), while both ADF and TRF regimens preserved catalase activity under irradiation. Notably, TRF-irradiated rats had significantly higher catalase activity than ADF-irradiated rats (25.6, *p* = 0.0241). For GSH, TRF-irradiated rats exhibited significantly higher levels than NF-irradiated rats (2.5, *p* < 0.0001), and although ADF-irradiated rats also had higher GSH than NF-irradiated, this difference was not statistically significant (0.6, *p* = 0.3124). Additionally, TRF-irradiated rats showed significantly higher GSH than ADF-irradiated rats (1.8, *p* < 0.0001), indicating a more robust effect of TRF in suggesting antioxidant capacity. Regarding GST, TRF-irradiated rats had significantly higher activity than NF-irradiated rats (0.05, *p* = 0.0002), while the increase in ADF-irradiated rats versus NF-irradiated was not significant (0.01, *p* = 0.4563). TRF-irradiated rats also had higher GST activity than ADF-irradiated rats (0.03, *p* = 0.0224). Collectively, these findings indicate that both ADF and TRF regimens mitigated radiation-induced impairment of antioxidant enzymes, with TRF generally providing suggesting protection (Fig. 3c–e).

Collectively, these findings demonstrated that IF regimens, especially TRF, not only suppress radiation-induced oxidative stress but also enhance the resilience of endogenous antioxidant mechanisms.

### Protective Effects of Intermittent Fasting (ADF and TRF) on Radiation-Induced Alterations in Kidney and Liver Function Biomarkers

Radiation exposure significantly impaired renal and hepatic function, as demonstrated by alterations in serum biomarkers. For renal function, two-way ANOVA showed significant main effects of diet (*F*(2, 24) = 14.6, *p* < 0.0001) and radiation (*F*(1, 24) = 342.6, *p* < 0.0001) on serum urea levels, with no significant interaction (*F*(2, 24) = 2.1, *p* = 0.1452). Irradiated rats on NF regimen exhibited markedly elevated urea concentrations compared to non-irradiated NF (38.4 mg/dL, *p* < 0.0001). Both ADF and TRF significantly attenuated this increase, with TRF-irradiated animals showing a greater reduction in urea levels compared to ADF-irradiated rats (*p* = 0.0138). In contrast, serum creatinine demonstrated a significant interaction between diet and radiation (*F*(2, 24) = 63.1, *p* < 0.0001), and a significant main effect of diet (*F*(2, 24) = 108.4, *p* < 0.0001), but no main effect of radiation alone (*F*(1, 24) = 1.2, *p* = 0.2926). NF-irradiated rats had significantly higher creatinine levels than NF non-irradiated controls (0.51 mg/dL, *p* < 0.0001). Both ADF and TRF markedly reduced creatinine in irradiated animals, with TRF showing superior efficacy over ADF (*p* = 0.0120), indicating enhanced renal protection by TRF.

Hepatic function markers ALT, AST, and GGT were all significantly affected by radiation and diet, with significant interactions observed for ALT (*F*(2, 24) = 29.6, *p* < 0.0001), AST (*F*(2, 24) = 3.7, *p* = 0.0383), and GGT (*F*(2, 24) = 38.8, *p* < 0.0001). Radiation significantly increased ALT activity in NF rats compared to controls (17.9 U/L, *p* < 0.0001), with both ADF and TRF attenuating this increase; TRF-irradiated animals showed significantly lower ALT than ADF irradiated rats (*p* = 0.0370). Similarly, AST levels were elevated in NF irradiated rats (46.07 U/L, *p* < 0.0001) and were significantly reduced by both fasting regimens, with no significant difference between ADF and TRF in irradiated groups, indicating comparable hepatoprotection. GGT activity exhibited the most pronounced radiation-induced elevation (1.02 U/L, *p* < 0.0001) and was significantly lowered by ADF and TRF in irradiated animals; TRF-irradiated rats had significantly lower GGT than ADF-irradiated rats (*p* = 0.0007), highlighting a stronger hepatoprotective effect of TRF. No significant differences were observed among non-irradiated groups across diets for any renal or hepatic markers, confirming that intermittent fasting did not adversely affect baseline organ function (Table 3).

**Table 3:** Effect of intermittent fasting (ADF and TRF) on levels of serum biochemical markers on radiation-induced alterations in male rats

	Non-irradiated				Irradiated	
	NF	ADF	TRF	NF	ADF	TRF
Urea (mg/dl)	41.3 ± 2.6	33.9 ± 3.1^b^	31.1 ± 2.5^b^	79.7 ± 2.5^a^	76.0 ± 1.7^a^	63.1 ± 1.9^abc^
Creatinine (mg/dl)	1.1 ± 0.02	1.0 ± 0.04^b^	1.0 ± 0.04^b^	1.7 ± 0.07^a^	0.8 ± 0.01^ab^	0.6 ± 0.03^abc^
ALT (U/L)	52.3 ± 1.8	51.7 ± 1.1^b^	47.9 ± 1.9^b^	70.2 ± 0.9^a^	49.2 ± 1.2^ab^	41.4 ± 2.4^abc^
AST (U/L)	99.6 ± 2.4	94.7 ± 5.7^b^	93.5 ± 6.8^b^	145.7 ± 1.8^a^	120.2 ± 1.8^ab^	119.3 ± 4.2^ab^
GGT (U/L)	1.4 ± 0.03	1.3 ± 0.03^b^	1.2 ± 0.02^b^	2.4 ± 0.07^a^	1.7 ± 0.05^ab^	1.4 ± 0.05^abc^

*ALT* alanine aminotransferase, *AST* aspartate aminotransferase, *GGT* γ‑glutamyl transpeptidase; Data were presented as mean ± SE (*n* = 6). Data were statistically analyzed using a two-way analysis of variance (ANOVA) followed by a post hoc Tukey test

^a^Significant vs the NF group

^b^Significant vs the NF-irradiated group

^c^Significant between ADF-irradiated and TRF-irradiated groups at *p* < 0.05

Together, these results demonstrated that radiation induced significant renal and hepatic dysfunction, which is effectively mitigated by IF regimens, particularly TRF, which consistently provided protection by reducing urea, creatinine, and liver enzyme elevations, thereby preserving kidney and liver function under radiation stress.

### Effects of Intermittent Fasting (ADF and TRF) on Radiation-Induced Changes in Oxidative Stress and Metabolic Regulation via GFAP, 8-OHdG, IRS1, AKT1, and PI3 K Pathways in Rats

**Fig. 4:**
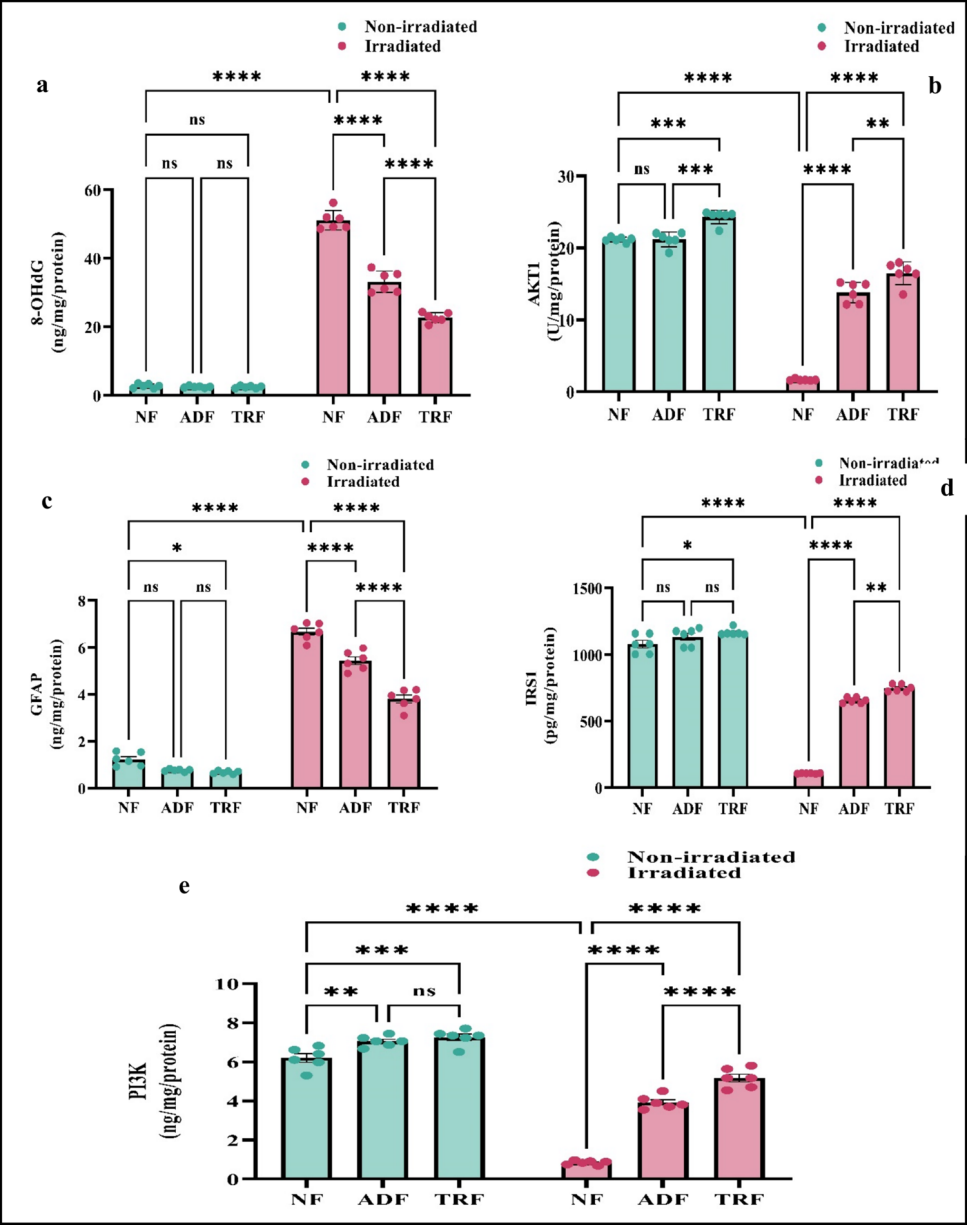
Intermitted fasting amends radiation-induced alterations in oxidative stress and metabolic biomarkers **a** 8-OHdG, **b** AKT1, **c** GFAP, **d** IRS1, and **e** PI3 K in the brain tissues of rats. Each bar with a vertical line represents the mean ± SE (*n* = 6). Statistical analysis was performed using two-way ANOVA followed by Tukey multiple comparison test at *p* < 0.05. ns: non-significant; *: significant at *p* < 0.05 level; **: significant at *p* < 0.01 level; ***: significant at *p* < 0.001 level; and****: significant at *p* < 0.0001 level

Two-way ANOVA was employed to evaluate the effects of dietary interventions and radiation exposure on key markers of oxidative stress and metabolic regulation in rats (Fig. 4).

Analysis of 8-OHdG, a biomarker of oxidative DNA damage, revealed highly significant main effects of diet (*F*(2, 30) = 185.6, *p* < 0.0001) and radiation (*F*(1, 30) = 2892, *p* < 0.0001), as well as a robust interaction between these factors (*F*(2, 30) = 177.8, *p* < 0.0001). Post hoc comparisons demonstrated a pronounced elevation of 8-OHdG in the NF-irradiated group relative to all other groups (*p* < 0.0001). Importantly, both ADF and TRF significantly attenuated radiation-induced increases in 8-OHdG, with TRF exhibiting a superior reduction compared to ADF (*p* < 0.0001), indicating a potent protective effect of TRF against oxidative DNA damage (Fig. 4a).

Building upon these findings, the investigation of AKT1—a pivotal kinase in cellular survival and metabolic pathways—also revealed significant main effects of diet (*F*(2, 30) = 230.4, *p* < 0.0001) and radiation (*F*(1, 30) = 1100, *p* < 0.0001), alongside a significant interaction (*F*(2, 30) = 129.4, *p* < 0.0001). AKT1 expression was markedly suppressed in the NF-irradiated group compared to all other cohorts (*p* < 0.0001). Notably, both fasting regimens restored AKT1 levels in irradiated animals, with TRF demonstrating a more pronounced restorative effect than ADF (*p* = 0.0015), underscoring its possiple efficacy in preserving metabolic signaling under radiation-induced stress (Fig. 4b).

Concomitantly, GFAP, an established marker of astrocyte activation and neuroinflammation, exhibited significant main effects of diet (*F*(2, 30) = 95.4, *p* < 0.0001) and radiation (*F*(1, 30) = 1918, *p* < 0.0001), with a significant interaction effect (*F*(2, 30) = 45.2, *p* < 0.0001). Radiation significantly elevated GFAP levels in NF animals (*p* < 0.0001), indicative of a suggestive neuroinflammatory response. Both ADF and TRF significantly mitigated this elevation, with TRF achieving a significantly greater reduction than ADF (*p* < 0.0001), highlighting its possible neuroprotective capacity (Fig. 4c).

Extending these observations to IRS1 was profoundly affected by radiation and diet, as evidenced by significant main effects of diet (*F*(2, 30) = 251.4, *p* < 0.0001) and radiation (*F*(1, 30) = 1934, *p* < 0.0001), and a significant interaction (*F*(2, 30) = 153.5, *p* < 0.0001). IRS1 levels were substantially diminished in NF-irradiated rats relative to NF (*p* < 0.0001). Both ADF and TRF significantly restored IRS1 expression in irradiated animals, with TRF exerting a significantly greater effect than ADF (*p* = 0.0089), suggesting enhanced preservation of insulin signaling pathways by TRF under radiation stress (Fig. 4d).

Finally, PI3 K, a key upstream regulator of AKT1 and metabolic homeostasis, was similarly influenced by diet and radiation, with significant main effects of diet (*F*(2, 30) = 156.1, *p* < 0.0001) and radiation (*F*(1, 30) = 749.0, *p* < 0.0001), and a significant interaction (*F*(2, 30) = 56.32, *p* < 0.0001). PI3 K expression was significantly reduced in NF-irradiated rats compared to non-irradiated NF (*p* < 0.0001). Both fasting protocols significantly ameliorated this reduction, with TRF demonstrating higher efficacy over ADF (*p* < 0.0001), reinforcing its role in possible maintaining metabolic signaling integrity following radiation exposure (Fig. 4e).

Collectively, these data provided compelling evidence that radiation induces significant oxidative damage and disrupts critical metabolic signaling pathways, while IF particularly TRF robustly counteracts these deleterious effects. The efficacy of TRF across multiple biomarkers underscores its potential as a strategic dietary intervention to mitigate radiation-induced oxidative stress and metabolic dysregulation.

### Effects of Intermittent Fasting (ADF and TRF) on Radiation‑Induced Alterations in mRNA Expression of BDNF and TrkB in Rats

**Fig. 5:**
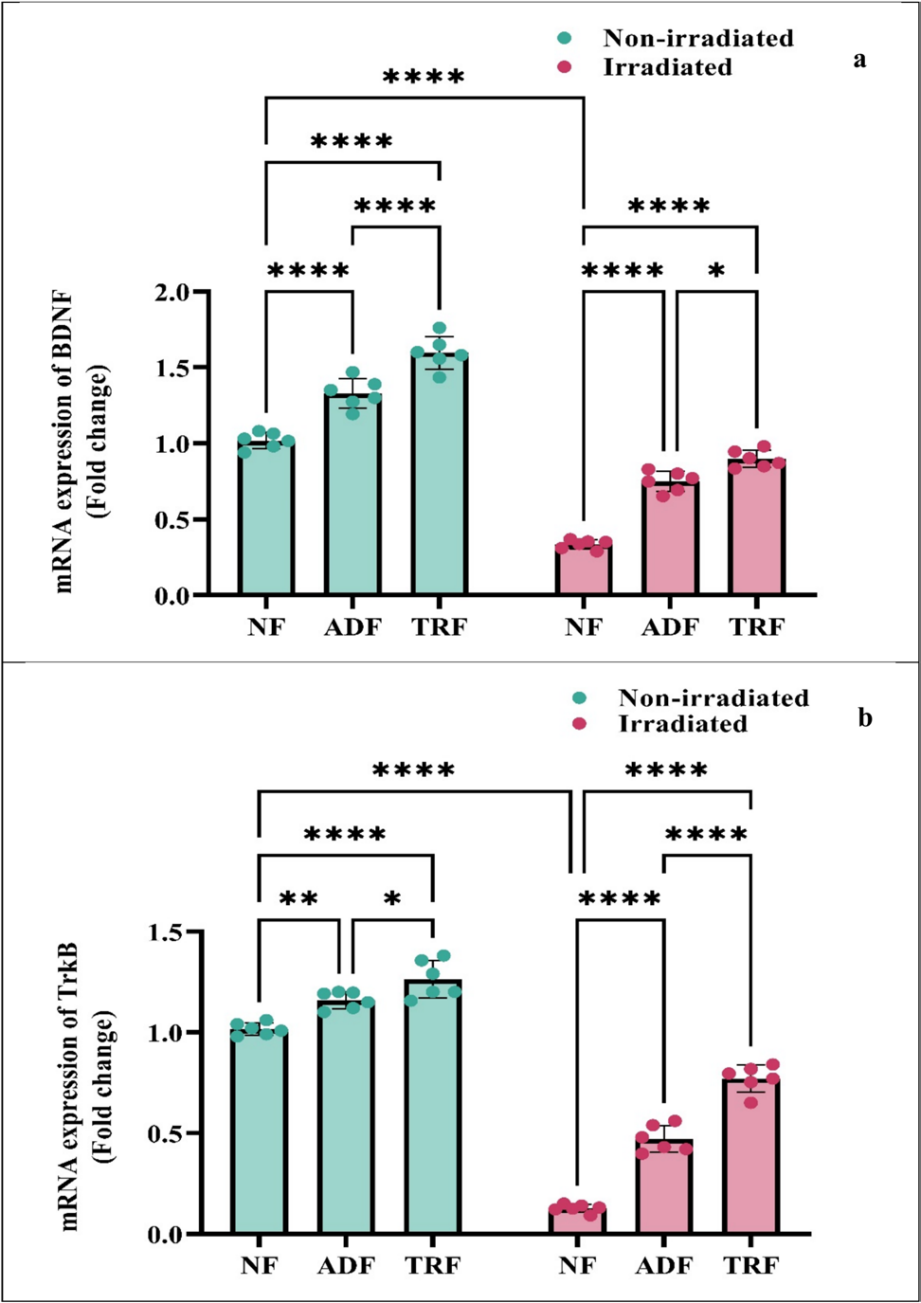
Intermitted fasting amends radiation-induced alterations in mRNA expression levels of **a** brain-derived neurotrophic factor (BDNF) and **b** tropomyosin receptor kinase B (TrkB) in rats. Each bar with a vertical line represents the mean ± SE (*n* = 6). Statistical analysis was performed using two-way ANOVA followed by Tukey multiple comparison test at *p* < 0.05. ns: non-significant; *: significant at *p* < 0.05 level; **: significant at *p* < 0.01 level; ***: significant at *p* < 0.001 level; and****: significant at *p* < 0.0001 level

The influence of dietary interventions and radiation exposure on TrkB and BDNF mRNA expression was evaluated using a two-way ANOVA (Fig. 5a), for BDNF expression was also significantly affected by diet (*F*(2, 30) = 185.8, *p* < 0.0001) and radiation (*F*(1, 30) = 716.0, *p* < 0.0001), though no significant interaction was observed (*F*(2, 30) = 2.3, *p* = 0.1137), suggesting that diet and radiation independently influenced BDNF levels. Radiation significantly reduced BDNF expression in NF-irradiated rats compared to NF non-irradiated (0.7, *p* < 0.0001). Both ADF and TRF significantly increased BDNF expression in irradiated animals (ADF-irradiated and TRF-irradiated) relative to NF-irradiated rats (*p* < 0.0001), while also enhancing BDNF levels in non-irradiated animals (ADF and TRF) compared to NF controls (*p* < 0.0001). TRF demonstrated a significantly greater restorative effect than ADF in irradiated rats (*p* = 0.0169), highlighting its possible enhanced capacity to mitigate radiation-induced neurotrophic deficits.

In parallel, TrkB, there were significant main effects of diet (*F*(2, 30) = 174.0, *p* < 0.0001) and radiation (*F*(1, 30) = 1248, *p* < 0.0001), alongside a significant interaction between these factors (*F*(2, 30) = 34.3, *p* < 0.0001), indicating that the effect of diet on TrkB expression depended on radiation status. Radiation caused a pronounced suppression of TrkB levels in NF rats compared to non-irradiated controls (0.9, *p* < 0.0001). Both ADF and TRF significantly reversed this decrease, with irradiated rats under these regimens showing markedly elevated TrkB expression relative to NF-irradiated rats (*p* < 0.0001). Furthermore, ADF and TRF increased TrkB expression in non-irradiated animals compared to NF controls (*p* = 0.0025 and *p* < 0.0001, respectively). Notably, TRF was more effective than ADF in restoring TrkB expression in irradiated rats (*p* < 0.0001), underscoring its possible neuroprotective potential (Fig. 5b).

### Histological Analysis of Cerebral Cortex and Hippocampal Tissues in Response to Alternate Day Fasting and Time-Restricted Feeding in a Rat Model of Radiation Exposure

#### Cerebral Cortex

The cerebral cortex tissue sections of the NF, ADF, and TRF groups showed normal histological structure without any significant pathological alterations. The cerebral cortex consisted of several layers of neuronal cells arranged with no sharp boundaries in association small blood vessels in between. The neuronal cells contained oval or rounded nuclei surrounded by scanty basophilic cytoplasm score (1).

**Fig. 6:**
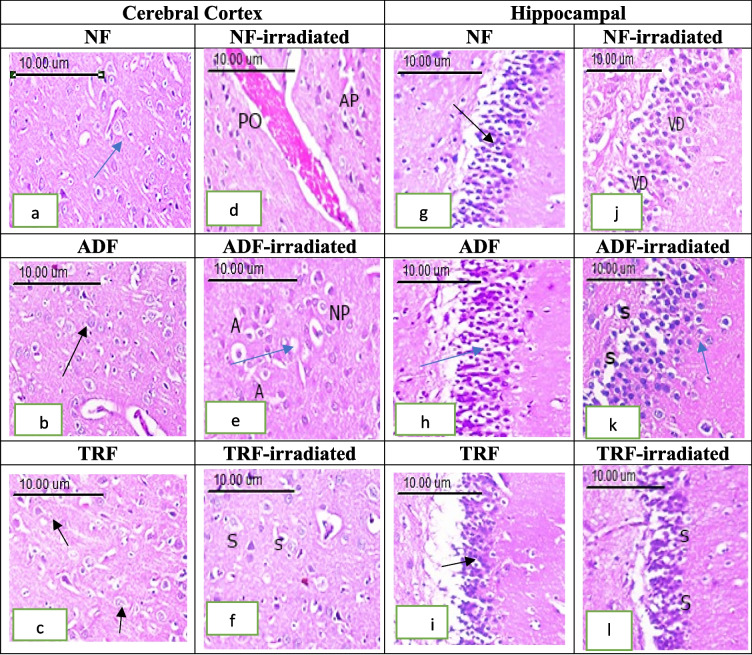
Photomicrograph of brain tissue section showing **a**–**c** normal histological structure of the cerebral cortex, **d** perivascular edema (PO) and apoptosis (AP) of neuronal cells, **e** apoptosis of neuronal cells (A) with nuclear pyknosis (NP), **f** mild swelling of neuronal cells (S), **g**–**i** normal layers of compact granular cells with dark nuclei in the dentate gyrus, **j** disorganization of neuronal cells and vacuolar degeneration (VD) of granular cell layers, **k** cellular disorganization and shrinkage (S) of pyramidal cells, **l** cellular organization mild swelling of granular cells (S), (H&E scale bar = 10.00 µm)

**Fig. 7:**
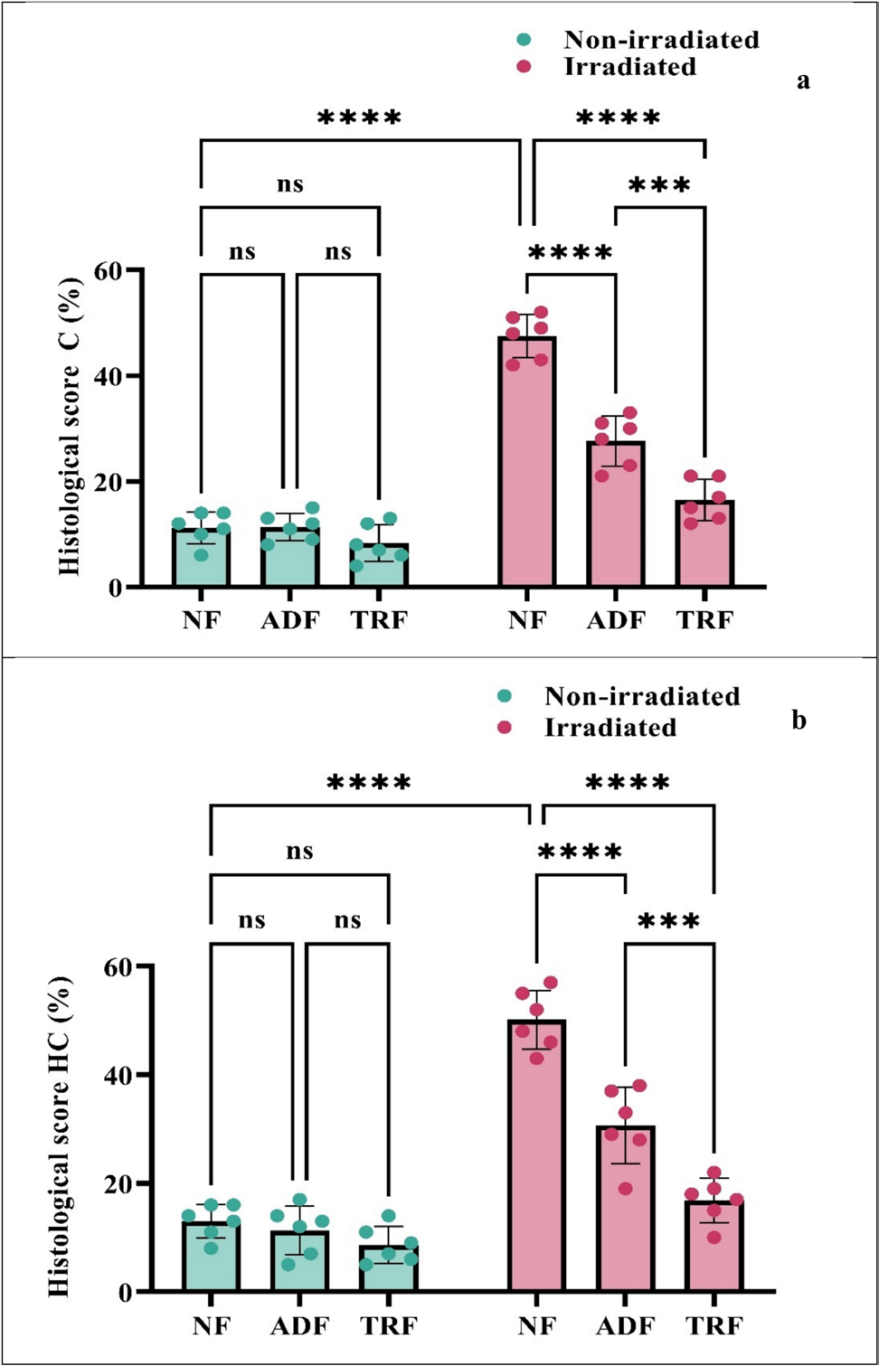
**a** Histological scores percentages of cortical damage (C).** b)** Histological scores percentages of hippocampal damage (HC) across treatment groups. Each bar with a vertical line represents the mean ± SE (*n* = 6). Statistical analysis was performed using two-way ANOVA followed by Tukey multiple comparison test at *p* < 0.05. ns: non-significant; *: significant at *p* < 0.05 level; **: significant at *p* < 0.01 level; ***: significant at *p* < 0.001 level; and****: significant at *p* < 0.0001 level

These findings were reflected in the summed cortical damage scores, which were 11.1 ± 1.2 for the NF group, 11.3 ± 1.0 for the ADF group, and 8.3 ± 1.4 for the TRF group, indicating preserved cortical integrity in non-irradiated animals, as shown in Fig. 6a–c and Fig. 7a.

In contrast, the radiation group exhibited significant pathological changes in the cortex, including shrunken, pyknotic neuronal nuclei, apoptotic cells with eosinophilic bodies surrounded by halo zones, congestion, perivascular edema, and gliosis. A tissue section of the cerebral cortex of an adult male albino rats exposed to radiation (NF-irradiated group) showed shrunken and darkly pyknotic neuronal nuclei. Apoptosis of neuronal cells which appeared as densely eosinophilic bodies surrounded by hallo zone. Congestion with perivascular oedema and gliosis were also detected score (3). These changes resulted in a markedly elevated cortical damage score of 47.5 ± 1.6, indicating substantial neurodegeneration following irradiation (Fig. 6d and Fig. 7a).

The ADF-irradiated group showed mild swelling of neuronal cells with few numbers of pyknotic nuclei. Gliosis and perivascular edema were also detected score (2). The summed cortical damage score in this group was 27.6 ± 1.9, suggesting partial neuroprotection by the ADF regimen (Fig. 6e and Fig. 7a).

On the other hand, the TRF-irradiated group demonstrated only mild neuronal degeneration, characterized by moderate swelling and focal gliosis score (1). This group had the lowest damage score among the irradiated groups, with a summed score of 16.5 ± 1.5, indicating near-complete preservation of cortical structure and a potentially stronger neuroprotective effect of TRF, as presented in Fig. 6f and Fig. 7a.

Two-way ANOVA analysis of cortical scoring revealed significant main effects of diet (*F*(2, 30) = 63.1, *p* < 0.0001) and radiation exposure (*F*(1, 30) = 269.6, *p* < 0.0001), as well as a significant interaction between these factors (*F*(2, 30) = 45.8, *p* < 0.0001). Radiation significantly increased cortical damage scores in NF rats, with NF-irradiated animals showing markedly higher scores compared to non-irradiated NF controls (36.3, *p* < 0.0001). Both ADF and TRF significantly reduced radiation-induced cortical damage, as irradiated rats under these dietary regimens exhibited lower scores relative to NF-irradiated rats (ADF: 16.50, *p* < 0.0001; TRF: 31.00, *p* < 0.0001). While no significant differences were observed among non-irradiated groups, TRF conferred a greater protective effect than ADF in irradiated animals, as indicated by significantly lower cortical scores (11.17, *p* = 0.0002). These results demonstrate that IF, particularly TRF, effectively mitigated radiation-induced cortical injury and suggested its potential as a neuroprotective strategy.

#### Hippocampus

In the hippocampus, histological evaluation of neuronal cell damage was conducted in four regions: CA1, CA2, CA3, and dentate gyrus (DG). Hippocampal section of dentate gyrus region of the NF, ADF, and TRF groups showed normal layers of compact granular cells with dark nuclei in dentate gyrus. Molecular layer showed glial cells as well as pyramidal cells score (1). The NF group showed mild baseline cell damage across all four hippocampal regions, resulting in a summed score of 13 ± 1.2. The ADF group had a summed score of 11.3 ± 1.8, while the TRF group recorded a lower summed score of 8.6 ± 1.3 **(**Fig. 6g–i and Fig. 7b).

Histological section of hippocampal neuronal cell damage in the radiation group (NF-irradiated) was evaluated and quantified in four regions of the left hippocampus, in H&E-stained section: CA1, CA2, CA3, and dentate gyrus (DG). When comparing individual hippocampal regions, it became apparent that the more affected region (DG) was generally more intensely damaged compared to the more lateral located CA1, CA2, and CA3 regions. A single high-power field (HPF) was focused on the DG region. Apoptotic neurons were clearly demarcated in the cell layers of all four assessed hippocampal regions. The DG region demonstrated cellular disorganization and reduction in numbers pyramidal cells with marked nuclear pyknosis was seen. Vacuolar degeneration of granular cell layers was also noticed score (3). This resulted in a summed score of 50.1 ± 2.2 (Fig. 6j and Fig. 7b).

Hippocampal (DG) region in the ADF-irradiated group revealed cellular disorganization and shrinkage of pyramidal cells, with darkened nuclei seen. Granular cell layers also showed vacuolar degeneration score (2), and a summed score of 30.6 ± 2.8, indicating a protective effect of ADF against radiation exposure (*p* < 0.0001) (Fig. 6k and Fig. 7b).

Histological section of hippocampal (DG) region in the TRF-irradiated group revealed cellular organization and vacuolar degeneration of neuronal cells score (1), resulting in a summed score of 16.8 ± 1.6, which was significantly lower than the NF-irradiated group (*p* < 0.0001) (Fig. 6l and Fig. 7b).

Two-way ANOVA analysis of hippocampal scoring revealed significant main effects of diet (*F*(2, 30) = 47.07, *p* < 0.0001) and radiation exposure (*F*(1, 30) = 184.0, *p* < 0.0001), along with a significant interaction between these factors (*F*(2, 30) = 28.25, *p* < 0.0001). Radiation substantially increased hippocampal damage scores in NF rats, with NF-irradiated animals showing significantly higher scores compared to non-irradiated NF controls (37.17, *p* < 0.0001). Both ADF and TRF significantly attenuated radiation-induced hippocampal damage, as irradiated rats under these dietary regimens exhibited lower damage scores relative to NF-irradiated rats (ADF, 19.50, *p* < 0.0001; TRF, 33.33, *p* < 0.0001). No significant differences were observed among non-irradiated groups, indicating that diet alone did not affect hippocampal scoring in the absence of radiation. Importantly, TRF demonstrated a greater protective effect than ADF in irradiated animals, as reflected by significantly lower hippocampal scores (13.83, *p* = 0.0003). These findings suggested that IF, particularly TRF, mitigated radiation-induced hippocampal injury and supported its possible potential as a neuroprotective intervention.

In summary, the analysis of the cerebral cortex and hippocampus revealed that the hippocampus was generally more severely affected by radiation compared to the cortex, particularly in the DG region. The protective effects of ADF and TRF were more pronounced in the hippocampus, with both ADF-irradiated and TRF-irradiated groups showing significantly lower summed scores compared to the NF-irradiated group, indicating neuroprotection. The comparison within each brain region further highlighted the differences between the treatment groups, with TRF demonstrating stronger protective effects than ADF.

## Discussion

As a non-medical dietary intervention, IF possesses promising capabilities in enhancing brain functions and emerging resistance to different types of brain insults [46]. The findings of this study suggest that intermittent fasting may exert protective effects against radiation-induced brain damage in a rat model, potentially through modulation of the IRS-1/PI3 K/AKT pathway and with possible contribution from BDNF/TrkB signaling.

It is well established that the BDNF/TrkB system contributes to the maintenance of neuronal cells, synaptic function, and neuroprotection. Therefore, the downregulation of the BDNF/TrkB system is critical for the pathophysiology of brain diseases [17]. A previous study indicated that whole-brain exposure to a 30-Gy dose of ionizing radiation revealed insult inhibition of mRNA and protein levels of BDNF and TrkB associated with induced cognitive dysfunction in rats [47].

In the same contest, our findings indicated that, cranial exposure to 20-Gy dose ionizing radiation caused increment inhibition of BDNF and TrkB gene expression compared with the control group which was in agreement with Liu et al. [48] who postulated that radiation induced BDNF-TrkB signaling dysregulation and decreased the levels of neuron-related functional genes which subsequently led to damage of neuronal ultrastructure and dendrites, loss of dendritic spines, and decline in the dendritic complexity index, contributing to spatial learning and memory deficits. Contradictory, our observation in groups under ADF and TRF showed a significant promotion of the mRNA expression of BDNF and TrkB levels in irradiated rats, suggesting that IF may affect the synthesis and secretion of BDNF and influence the BDNF-TrkB pathway in response to radiation exposure. There is potential for a positive influence of IF on BDNF and cognitive function [49].

There is strong evidence to support the increased production of BDNF as one of the most significant neuronal adaptations to IF [50]. Food deprivation induces a metabolic switch in neurons that increases excitatory synaptic activity, provoking calcium influx through membrane channels and leading to stimulation of a cascade of kinases and signaling pathways that in turn cause the expression of various genes that eventually code for proteins involved in cellular stress adaptation, one of which is BDNF [51]. The proper explanation of the effect of IF on BDNF gene expression may depend on the increased production of Β-hydroxybutyrate (BHB) during acute fasting periods [52]. Previous research illustrated that Β-hydroxybutyrate (BHB) induces BDNF gene expression in hippocampal and cortical neurons in cell culture and in vivo [53].

The increased excitatory synaptic activity associated with food deprivation does lead to elevated calcium influx, which can raise concerns about potential neurotoxic effects, similar to those observed in epilepsy. However, it is important to note that during fasting, the body undergoes a metabolic switch characterized by ketogenesis, which produces ketone bodies that serve as an alternative energy source for neurons. This shift not only improves energy supply but also has protective effects against oxidative stress, apoptosis, and inflammation. Ketone bodies, such as β-hydroxybutyrate, have been shown to enhance mitochondrial function and activate cellular protective mechanisms, thereby mitigating the potential neurotoxic effects of increased excitatory activity [54]. Specifically, they promote resistance to oxidative stress and inflammation, which are critical factors in neuronal health [55]. This adaptive response helps neurons cope with the metabolic challenges posed by food deprivation while maintaining cellular integrity.

One of the possible mechanisms by which BHB enhances BDNF production has been described by Sleiman et al. [56], potentially through the upregulation of BDNF gene transcription by inhibiting histone deacetylase, which normally represses BDNF expression. In agreement with our findings, it has been illustrated how IF improved memory and spatial learning by triggering the expression of BDNF and inducing an increased dendritic spine density in hippocampal dentate granule neurons [57]

Besides the protective neurotrophic effect of BDNF in brain tissue, it has been shown that BDNF can reduce neuroinflammation [58], which is an important determinant in neurodegenerative diseases [59]; therefore, we may postulate that IF may exhibit anti-inflammatory activity through aggravated activation of BDNF expression. Our findings suggest that the regulation of IF on the BDNF-TrkB pathway is responsible for its neuroprotective effect on radiation-induced brain impairment.

Also, the BDNF/TrkB system triggers numerous intracellular signaling such as ERK and PI3 K/Akt cascades [17]. The recognized and established neuro-signaling pathway involves the binding of insulin to its specific receptor, causing phosphorylation of insulin receptor substrate 1 (IRS-1) [60], leading to the activation of IRS-1 that results in downstream phosphorylation of PI3 K, and protein kinase B (PkB/Akt) [61].

Numerous investigations have revealed that damaged brain cells have markedly low levels of p-PI3 K and p-Akt. According to Endo et al. [62], ischemic neuronal death was exacerbated by inhibition of the PI3 K/Akt pathway. Yong et al. [63] demonstrated that the mRNA expression and protein expression levels of PI3 K and AKT in radiation-exposed rats were suppressed and associated with active neuronal apoptosis and induced brain injury.

Therefore, it may be suggested that IF could exhibit anti-inflammatory activity through the regulation of BDNF expression. Our findings indicated that the regulation of IF on the BDNF-TrkB pathway may play a role in its effects on radiation-induced brain impairment. In contrast, triggering the PI3 K/Akt signaling pathway may help recover from acute brain injury [64, 65].

Furthermore, β-hydroxybutyrate (βHB) production is known to increase during acute fasting periods [52], which in turn causes an increase in IRS1 and Akt phosphorylation in their active forms [66]. In the current study, IF increased the expression of IRS-1/PI3 K/Akt; therefore, we can postulate that IF is capable of accessing brain health via regulating metabolism, neuronal growth, proliferation, and other cellular functions. This study agrees with previous studies [66–68].

Our study demonstrated that IF elicits differential responses in various organs when exposed to radiation. Research indicates that distinct tissues have varying sensitivities to radiation, largely influenced by factors like cell turnover rates and metabolic activity. Rapidly dividing tissues, such as bone marrow and gastrointestinal mucosa, are particularly susceptible to acute radiation damage due to their high proliferative activity. In contrast, organs like the liver and brain may exhibit delayed responses characterized by oxidative stress and inflammation [69, 70]. Our findings suggest that IF not only enhances neuroprotection in the brain but also positively influences metabolic pathways in peripheral organs, indicating systemic protective effects against radiation-induced damage. The potential modulation of the IRS-1/PI3 K/AKT and BDNF/TrkB signaling pathways may play a role in the neuroprotective effects of intermittent fasting against radiation-induced damage. These pathways are known to support cellular survival and reduce apoptosis in response to stressors such as radiation.

Literature supports our findings, indicating that these pathways can mitigate oxidative stress and enhance neurogenesis following radiation injury [71, 72]. Increased levels of BDNF have been associated with improved cognitive function and neuronal survival after radiation exposure, underscoring the importance of these signaling pathways in mediating IF’s protective effects.

Moreover, GFAP is a particularly brain-specific protein [73]. GFAP is typically used as a marker of astroglial stimulation owing to its marked elevated expression in a wide range of pathological illnesses, including neuronal injuries and degeneration [74]. Various neurodegenerative and non-neurodegenerative neurological disorders have been linked to elevated blood GFAP concentrations [75].

Tian et al. [76] demonstrated that GFAP mRNA was upregulated in the hippocampus as early as 24 h after 10 and 30 Gy of radiation exposure, whereas a marked increase in GFAP-positive astrocytes was observed 1 month after 30-Gy irradiation suggesting its crucial role in the mechanisms underlying radiation-induced brain damages in the early stages. Also, according to Monje et al. [77], the increase in GFAP 2 weeks after radiation exposure is associated with microglial cell proliferations, which secrete cytokines that change cell fate in the dentate gyrus. It is believed that persistent neuroinflammation probably modifies neural progenitor’s functions and their interactions with the adjacent glial, neurons, and vascular cells in the local microenvironment. These observations are consistent with our finding on the effect of 20-Gy cranial radiations with pronounced elevated levels of GFAP as a marker of brain injury.

Brain oxidative stress is closely associated with a diversity of neurodegenerative illnesses, stroke, and traumatic brain injury (TBI). ROS accumulation has a major role in the pathophysiology of these diseases and contributes to neuronal injury and loss. Furthermore, oxidative stress is also connected to blood–brain barrier dysfunctions and neuroinflammation, which are important aspects in the development of neurological illnesses [5].

ROS accumulation in the brain may cause oxidative damage to lipids, proteins, and DNA, resulting in loss and damage of neurons [78]. MDA serves as a marker for lipid peroxidation, indicating the degradation of cell membrane integrity due to oxidative stress. Elevated levels of MDA reflect the extent of lipid damage caused by free radicals. 8-OHdG, on the other hand, is a marker for oxidative DNA damage. Its elevation signifies increased oxidative stress leading to DNA base modifications, which can contribute to mutagenesis and cellular dysfunction [79]. Elevation of MDA, a lipid peroxidation marker, and 8-OHdG (a marker of DNA oxidation) indicate that free radicals can damage cell membranes and DNA.

It is well known that radiation induces oxidative stress, which can result in metabolic stress, damage of DNA, and further detrimental effects on biochemical processes in brain tissue [80]. These findings are consistent with our observed investigation. The current study indicated that ionizing radiation did not significantly affect body weight when compared to the normal feeding group (NF), while it stimulated GFAP protein expression, provoked the DNA damage marker 8-OHdG, and increased levels of oxidative damage and lipid peroxidation marker MDA with a depletion of the antioxidant content of GSH, GST, and CAT activities. Also, IR-induced renal and liver dysfunction manifested in elevated renal markers and liver enzyme activity**.** However, IF dramatically decreased body weight, GFAP protein expression, and MDA concentration, while significantly increasing GSH concentration in brain tissues; these results were also illustrated by other research indicating the pronounced IF mechanistic effect in altering oxidative changes and inhibition of GFAP protein expression in improving memory performance dysfunction [81].

Hu et al. [82] proposed a neuroprotective effect of short-term IF which can decrease the redox state and neuroinflammation. It has also been reported that post-operative IF reduces the concentration of MDA and increases the concentration of GSH in brain tissues. In agreement with these studies, the current study illustrated that IF improves brain structure and function by inhibiting oxidative damage. This finding could be attributed to the capability of IF to alleviate the radiation-induced oxidative stress state, which was indicated by a significant reduction in 8-OHdG and MDA levels with a significant increase in GSH content, GST, and CAT activities. Also, IF improved liver and kidney functions. This study agrees with previous studies [81–84].

Also, the current study demonstrated a positive association between histopathological, biochemical, and molecular investigations, indicating radiation-induced brain tissue damage with perivascular edema and apoptosis of neuronal cells cross-linking with the induced oxidative stress state, whereas IF restored brain neuronal cell health. The elucidated mechanism by which IF alleviates radiation damage may be through suppression of oxidative stress and activation of the expressions of BDNF/TrkB and IRS-1/PI3 K/Akt pathways, which are responsible for neuronal differentiation, cell survival, and synaptic function, leading to improved brain structure and function.

In this study, we have highlighted that the present results indicated a stronger effect of TRF on several neuroprotective outcomes compared to ADF. Previous studies demonstrated that TRF may lead to more consistent metabolic adaptations due to its structured feeding window, enhancing neuroprotective pathways. By limiting food intake to specific hours, TRF has been shown to improve insulin sensitivity, which is crucial for neuronal health and energy supply [85]. Additionally, TRF reduces oxidative stress by lowering the production of ROS, thus protecting neurons from damage [86]. Aligning food intake with circadian rhythms optimizes metabolic processes and enhances neuroprotective mechanisms [87]. Furthermore, TRF activates cellular stress response pathways, promoting resilience in neurons [51]. Collectively, these factors suggest that TRF is a promising strategy for enhancing brain health and resilience against stressors such as radiation.

While our study demonstrated significant associations between IF and neuroprotective outcomes against radiation-induced brain damage, it is important to clarify that these findings do not establish direct causality. The observed neuroprotective effects and associated modulation of key signaling pathways, such as BDNF and PI3 K, highlight the need for further investigation. Future studies using models like BDNF-deficient mice or pharmacological inhibitors of these pathways will be essential to clarify the causal relationships underlying the protective effects of intermittent fasting. While our findings contribute to the growing body of evidence suggesting that IF may enhance brain resilience [72, 88], a deeper mechanistic understanding is necessary to support the translation of these findings into clinical applications.

In the present study, we investigated the neuroprotective effects of intermittent fasting against radiation-induced brain damage, with a particular focus on the potential modulation of BDNF and PI3 K signaling pathways. While our findings suggest a possible association between IF and neuroprotection, we acknowledge the pivotal role of inflammation in these processes. Therefore, future research should include direct assessment of inflammatory markers, such as cytokines (e.g., IL-6, TNF-α), in both blood and brain tissues. This approach will help clarify the interplay between fasting, neuroinflammation, and radiation exposure, thereby advancing our understanding of the mechanisms underlying the neuroprotective effects of IF.

## Conclusions

The conclusion of this study highlights the efficacy of IF, specifically ADF and TRF, in mitigating oxidative stress and promoting neuroprotection against radiation-induced brain damage in rats. The observed stronger effects of TRF compared to ADF suggested that variations in fasting strategies can significantly influence brain health outcomes.

## Limitations

However, it is crucial to acknowledge limitations, including the reliance on animal models that may not fully replicate human responses to ionizing radiation, as well as the differences in radiation exposure protocols between studies and clinical settings. The discrepancy between the single, whole-body radiation exposure typically seen in animal studies and the more complex, fractionated, and partial-body irradiation regimens used in human radiotherapy poses a significant challenge. One significant constraint is the absence of organ-specific RT-PCR data. This limitation restricts our ability to fully elucidate how IF may differentially affect gene expression across various tissues, which is essential for understanding the systemic implications of our findings.

## Future Directions

Future research should focus on elucidating the specific molecular mechanisms involved, assessing the long-term effects of IF in human populations, and optimizing fasting regimens for clinical application. To build upon our current work, future research should focus on conducting organ-specific analyses to investigate the effects of IF on gene expression in various organs, particularly those involved in neuroprotection and metabolic regulation. Such studies could provide deeper insights into the mechanisms through which IF exerts its protective effects against radiation damage and may reveal potential therapeutic targets for mitigating radiation-induced injuries.

## Translational Avenues

Translational avenues could involve developing dietary strategies or pharmacological interventions that mimic IF’s protective effects, potentially offering new therapeutic options for patients undergoing.

## Data Availability

No datasets were generated or analysed during the current study.
